# Deciphering the mechanistic landscape of immune checkpoint blockade in ccRCC: from molecular drivers to therapeutic responses

**DOI:** 10.3389/fimmu.2026.1774959

**Published:** 2026-05-12

**Authors:** Lingxiang Ran, Guangmo Hu, Chunyu Fan, Yuanyin Teng, Rui Zhao, Qinghua Li, Jing-Min Yang, Chao Zhang

**Affiliations:** 1 Department of Urology, The First People’s Hospital of Hefei, Hefei, China; 2 Department of Urology, Peking University First Hospital, Beijing, China; 3 Department of Urology, The Second Affiliated Hospital of Soochow University, Suzhou, China; 4 West China Biomedical Big Data Center, West China Hospital, Sichuan University, Chengdu, China; 5 Institute of Hematology, Zhejiang University, Hangzhou, China; 6 Acupuncture Center, Yueyang Hospital of Integrated Traditional Chinese and Western Medicine, Shanghai University of Traditional Chinese Medicine, Shanghai, China

**Keywords:** artificial intelligence, immune checkpoint inhibitors, multi-omics data, renal cell carcinoma, resistance

## Abstract

Immune checkpoint inhibitor (ICI) based combination therapies have revolutionized the management of advanced clear-cell renal cell carcinoma (ccRCC), establishing a new standard of care and significantly improving survival outcomes. However, this success is challenged by substantial heterogeneity in patient response, with primary and acquired resistance remaining major clinical hurdles that limit durable benefit for a substantial proportion of patients. This review synthesizes our current understanding of the multifaceted mechanisms governing these outcomes. We explore the complex interplay between tumor-intrinsic drivers of resistance, such as mutations in key genes like PBRM1, and the profoundly immunosuppressive landscape of the tumor microenvironment (TME), which includes diverse inhibitory cell populations, metabolic reprogramming, and stromal barriers. We then highlight how multi-omics technologies, from single-cell and spatial transcriptomics to proteomics, are decoding the TME’s intricate cellular and spatial architecture to reveal novel biomarkers and therapeutic targets. Crucially, we discuss the pivotal role of artificial intelligence (AI) in translating this high dimensional data into clinically actionable insights. AI-driven models in pathomics and radiomics are creating powerful, non-invasive tools to predict treatment response and prognosis from images, while deep learning algorithms are proving essential for integrating multi-omics data to guide patient selection and accelerate drug discovery. Ultimately, the convergence of these advanced biological insights and computational strategies is paving the way for precision immuno-oncology, with the goal of moving beyond current risk stratification toward truly personalized ICI therapy for patients with ccRCC.

## 1 Introduction

Renal cell carcinoma (RCC) represents one of the most common malignancies of the urogenital tract, with an estimated 434,419 new cases and 155,702 deaths worldwide in 2022, based on the latest GLOBOCAN estimates (1). In the United States, approximately 80450 new cases of kidney and renal pelvis cancer and 15,160 deaths are projected for 2026 (2). The incidence of RCC has demonstrated an upward trend over the past few decades (3, 4). Clear-cell RCC (ccRCC) is the predominant histological subtype, accounting for approximately1 70–80% of all RCC cases, and is the primary focus of this review (5).

Prior to the advent of immune checkpoint inhibitors (ICIs), the prognosis for patients with metastatic RCC (mRCC) was poor, with historical data indicating a 5-year survival rate of merely 12%; for advanced ccRCC, this rate was even lower, at less than 10% (6–8). Early therapeutic strategies, such as cytokine therapies with interleukin-2 (IL-2) and interferon-α (IFN-α), confirmed the immunogenic potential of RCC. High-dose IL-2, in particular, yielded objective response rates (ORR) of around 20%, but these treatments were often accompanied by substantial toxicity (9, 10). Subsequently, targeted agents against the vascular endothelial growth factor (VEGF) and mammalian target of rapamycin (mTOR) pathways, notably VEGF tyrosine kinase inhibitors (TKIs), became the mainstay of mRCC treatment for over a decade. Although these drugs significantly improved progression-free survival (PFS), clinical practice revealed the development of both primary and acquired resistance to TKI monotherapy. Consequently, complete responses (CR) remained rare, and the majority of patients eventually experienced disease progression due to resistance (11–14).

The emergence of ICIs—monoclonal antibodies targeting programmed death receptor-1 (PD-1), its ligand PD-L1, and cytotoxic T-lymphocyte-associated antigen-4 (CTLA-4)—has revolutionized the treatment landscape of mRCC (15–17). By blocking these key immune checkpoint pathways that facilitate tumor immune evasion, ICIs effectively restore and augment T cell-mediated antitumor immunity. A landmark breakthrough in the field was the Phase III CheckMate 025 trial, in which Motzer R.J. et al. demonstrated that nivolumab monotherapy as a second-line treatment for mRCC offered an overall survival (OS) benefit and a more favorable safety profile compared to everolimus (18). Following this success, ICI-based combination therapies, including dual ICI regimens (for example, nivolumab plus ipilimumab) and ICI-TKI combinations (such as pembrolizumab plus axitinib or lenvatinib, and nivolumab plus cabozantinib), have rapidly been established as the first-line standard of care for patients with advanced RCC across different risk strata, leading to marked improvements in both PFS and OS (19–23). Furthermore, in the adjuvant setting, the positive results from the KEYNOTE-564 study showed that pembrolizumab improved disease-free survival (DFS) and demonstrated a significant OS benefit in patients with high-risk ccRCC following nephrectomy, thereby expanding the application of ICIs (9, 24–26).

Despite these remarkable achievements, the clinical application of ICIs in RCC faces substantial challenges. A central bottleneck limiting the efficacy and durability of ICI therapy is the development of primary and acquired resistance (27). Indeed, recent findings from studies such as CONTACT-03 and TiNiVo-2 suggest that for patients who progress on ICI therapy, subsequent ICI rechallenge strategies offer limited value and might even increase toxicity (28, 29). Moreover, a critical deficiency in current clinical practice is the lack of reliable predictive biomarkers to accurately identify patients who are most likely to benefit from a specific ICI regimen or to predict the risk of resistance, which severely hinders the implementation of personalized immunotherapy (27). This pronounced heterogeneity in treatment response strongly indicates that the efficacy of immunotherapy in RCC is not a one-size-fits-all paradigm but is instead governed by a complex interplay of tumor-intrinsic biological features, the composition of the tumor microenvironment (TME), and various host factors (27, 30). Therefore, elucidating the mechanisms of ICI resistance and developing novel, multi-dimensional predictive tools are among the most urgent scientific questions in the field of RCC immunotherapy.

Artificial intelligence (AI), particularly machine learning and deep learning, is emerging as a powerful tool to address these complexities, owing to its profound capabilities in data processing and pattern recognition (27, 31–33). AI has shown immense potential in medical image analysis, pathological assessment, integration of multi-omics data, and the development of sophisticated biomarker models. These applications offer unprecedented opportunities to deepen our understanding of ICI resistance mechanisms, discover novel predictive biomarkers, and optimize individualized treatment strategies (34–36).

This review aims to systematically summarize recent advances in the biological mechanisms, clinical research, resistance mechanisms, and corresponding management strategies related to ICI therapy in RCC. A key focus will be placed on the pivotal role and future prospects of AI in advancing personalized immunotherapy for ccRCC.

## 2 Results

### 2.1 Mechanisms of ICIs in ccRCC

The successful application of ICIs is rooted in a profound understanding of tumor immune evasion mechanisms; these agents function by reactivating the host’s immune system to combat cancer (37, 38). ICIs primarily act by blocking inhibitory checkpoint pathways that tumor cells exploit to escape immune destruction, with the canonical PD-1/PD-L1 and CTLA-4 axes serving as the core targets of current therapies (11, 19, 22, 24). PD-1 is predominantly expressed on the surface of activated T cells, B cells, and natural killer (NK) cells, whereas its ligands, PD-L1 and PD-L2, are widely expressed on various cell types, including tumor cells and antigen-presenting cells (APCs) (39–44). The engagement of PD-1 by PD-L1 suppresses T cell proliferation, activation, and cytokine production, leading to T cell exhaustion and enabling tumor cells to evade immune surveillance (39, 41). In contrast, CTLA-4 primarily functions as a negative regulator during the early stages of T cell activation. It competes with CD28 for binding to B7 molecules (CD80/CD86), thereby inhibiting the initial activation and proliferation of T cells (11, 19, 22, 24). Consequently, anti-PD-1/PD-L1 antibodies restore the cytotoxic function of T cells by disrupting the PD-1/PD-L1 signaling pathway, whereas anti-CTLA-4 antibodies mainly enhance T cell priming and activation within lymphoid organs (19, 22, 24, 39–42). However, the heterogeneous response of RCC to ICIs and the prevalence of resistance underscore that the mechanisms of immune escape are far more complex. In recent years, investigations into the dynamic regulation of the RCC TME, tumor metabolic reprogramming, and tumor-cell-intrinsic escape pathways have unveiled additional biological mechanisms and potential therapeutic targets that influence ICI efficacy (45, 46).

#### 2.1.1 Emerging immune checkpoints and pathways

**Figure 1:**
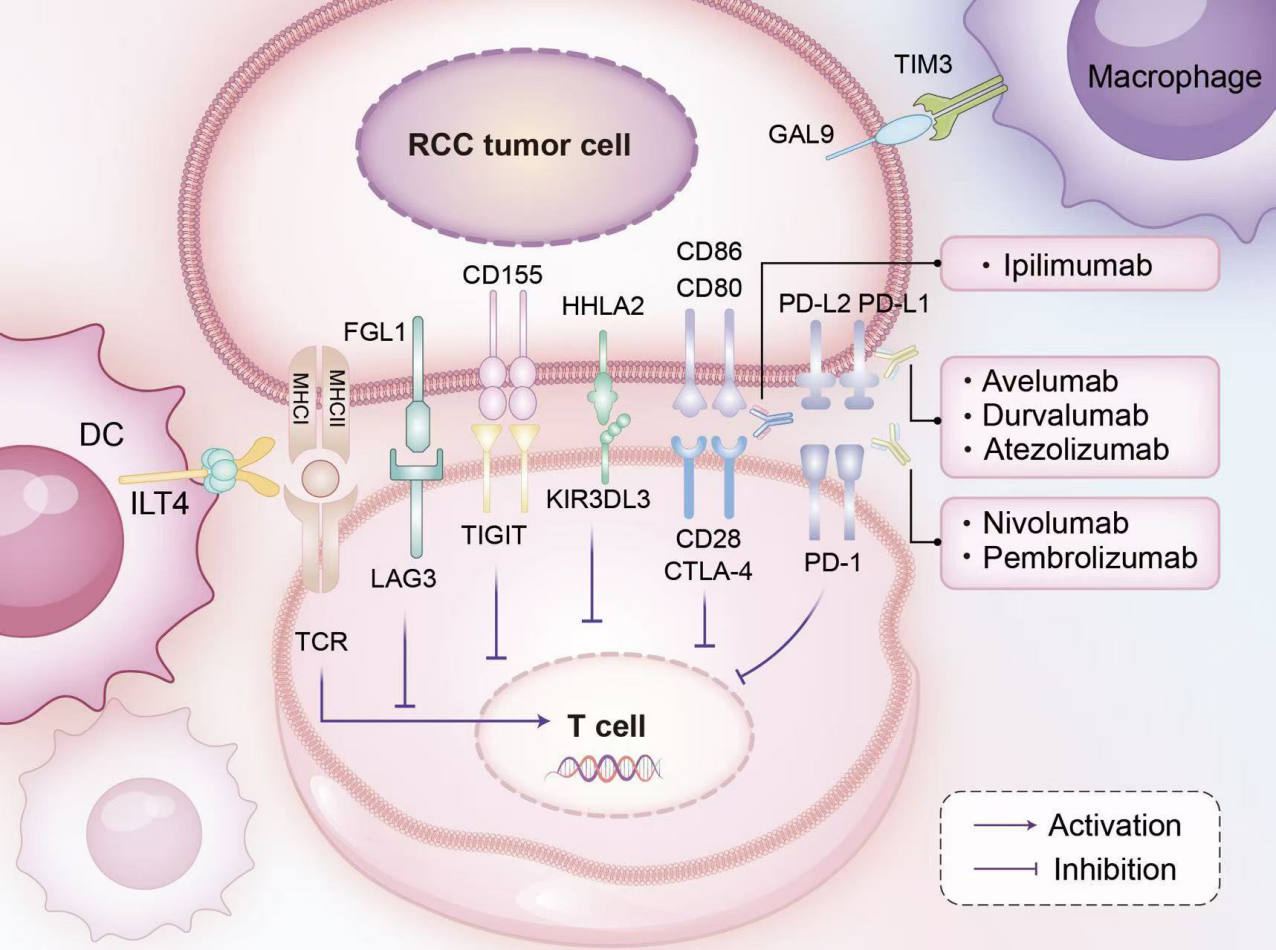
Mechanisms of immune checkpoint inhibitors (ICIs) in clear cell renal cell carcinoma (ccRCC). The signaling of immune checkpoint inhibitors (ICIs) is intricately regulated through interactions among various cells, including RCC tumor cells, T cells, macrophages, and dendritic cells. RCC tumor cells present tumor peptides to T cells via major histocompatibility complex (MHC) glycoproteins, engaging the T cell receptor (TCR). Key inhibitory interactions occur as PD-L1/2, CD80/CD86, CD115, and FGL1 on RCC tumor cells bind with PD-1, CD28/CTLA-4, TIGIT, and LAG3 on T cells, respectively; notably, HHLA2 on tumor cells interacts with KIR3DL3 on T cells. Additionally, RCC tumor cell-expressed GAL-9 and MHC1 interact with TIM3 on macrophages and ILT4 on dendritic cells, modulating immune responses. By blocking these inhibitory pathways, ICIs restore T cell function, enhance tumor cell recognition, and bolster anti-tumor immunity.

As our understanding of the PD-1/PD-L1 and CTLA-4 pathways has deepened and the challenge of resistance has become more apparent, researchers have shifted their focus to other targets, often termed ‘next-generation’ immune checkpoint molecules. Novel checkpoints such as LAG-3, TIGIT, and TIM-3 are frequently co-expressed with PD-1 on exhausted T cells, contributing to their dysfunction (47). Therefore, targeting these molecules, particularly in combination with PD-1/PD-L1 inhibitors, has emerged as a biologically compelling and promising strategy to overcome resistance to current immunotherapies, although its clinical benefit in ccRCC remains to be fully established. For instance, the LAG-3 inhibitor relatlimab, in combination with nivolumab, has been approved for the treatment of melanoma, showcasing its potential (48, 49). Specifically, LAG-3 downregulates the immune response by inhibiting T cell proliferation and function; in RCC, its expression is associated with immunotherapy response and improved survival in high-risk patients (47). TIGIT dampens immune responses by competitively binding to its ligands, such as CD155, thereby inhibiting the activation of T cells and NK cells (50); high expression of TIGIT has been observed in RCC tumor tissues (51). TIM-3, through its interaction with its ligand Galectin-9, induces Th1 cell exhaustion and promotes immune tolerance. Elevated TIM-3 expression is also observed in RCC, and targeting it holds promise for restoring T cell function (52, 53). Furthermore, inhibitory receptors expressed primarily on myeloid cells, such as immunoglobulin-like transcript 4 (ILT4), are being explored as therapeutic targets. ILT4 interacts with MHC class I molecules to foster an immunosuppressive TME (54), and its inhibitors have shown preliminary antitumor activity in advanced solid tumors (55). These emerging immune checkpoint molecules and their pathways are illustrated in **Figure 1**.

A notable example is the HHLA2 (B7-H7)/KIR3DL3 axis. HHLA2 (HERV-H LTR-associating protein 2), also known as B7-H7, is a member of the B7 ligand family that exhibits complex dual roles in immune regulation. KIR3DL3, an inhibitory receptor primarily expressed on T cells and NK cells, was recently identified as a key functional receptor for HHLA2 (56). Research by Bhatt et al. indicated that in ccRCC, the expression of HHLA2 and PD-L1 is often non-overlapping, suggesting that HHLA2 might mediate immune evasion through a pathway independent of PD-1/PD-L1 signaling. When HHLA2 on the surface of tumor cells binds to KIR3DL3 on immune cells, it recruits SHP-1/SHP-2 phosphatases via its ITIM motif. This recruitment suppresses downstream signaling pathways, including Vav1, ERK, AKT, and NF-κB, leading to a comprehensive inhibition of CD8+ T cell and NK cell functions (56, 57). The potential of combined HHLA2 and PD-L1 expression as a predictive biomarker for ICI therapy in ccRCC patients is currently being evaluated in a study related to the HCRN GU16–260 trial (58). Additionally, tumor-cell-intrinsic epigenetic dysregulation provides further clues for understanding resistance and identifying new targets. For instance, Chung et al. discovered a “SOX10→MITF→GPNMB” cascade activated by the PD-L1 signaling pathway in RCC with acquired ICI resistance (59), while the hypomethylation status of the CTLA4 gene promoter has been linked to ICI efficacy (20). However, recent clinical data suggest that not all emerging checkpoint strategies translate into superior efficacy in ccRCC. In the KEYMAKER-U03 substudy presented at ESMO 2025, LAG-3- and TIGIT-based investigational arms did not outperform the pembrolizumab plus lenvatinib reference arm, indicating that alternative resistance mechanisms may be more important therapeutic targets in some patients (60). The complexity and diversity of these mechanisms highlight the necessity for personalized therapeutic approaches.

#### 2.1.2 Dynamic regulation of the tumor microenvironment (TME)

The clinical success of ICIs is profoundly influenced by the dynamic regulation of the complex TME. Traditionally, the quantity of tumor-infiltrating lymphocytes (TILs), particularly CD8+ T cells, has been regarded as a key indicator for assessing antitumor immune responses and predicting ICI efficacy (61, 62). However, the TME is also widely populated by immunosuppressive cell types, such as regulatory T cells (Tregs) and myeloid-derived suppressor cells (MDSCs), which inhibit T cell activation and promote immune tolerance through various mechanisms, thereby substantially limiting the therapeutic effect of ICIs (63–65).

Recent in-depth studies of the TME in RCC, especially ccRCC, have further revealed its complexity and high degree of heterogeneity. First, the RCC TME exhibits significant molecular heterogeneity, allowing for its classification into distinct molecular subtypes, such as vascular/stromal and T-effector/proliferative types. These subtypes possess unique immune cell infiltration patterns and microenvironmental components, which are closely associated with patient response to ICIs (66). Second, the ‘quality’ and functional state of specific immune cell subsets, rather than their mere quantity, may be more critical for ICI efficacy. For example, the infiltration of an NKG2A(+)CD8(+) T cell subset is associated with an immunosuppressive microenvironment and poorer response to ICI therapy (23). Concurrently, an IL1B-expressing Treg subset identified in ccRCC may play a pivotal immunosuppressive role within the TME (30), while the expression patterns of effector T cell-related markers (such as the ratio of PD-1/CD8 and CD39/CD3) also serve as important prognostic factors (67). Furthermore, stromal components within the TME, such as cancer-associated fibroblasts (CAFs) and endothelial cells (ECs), display considerable sub-cluster heterogeneity and unique spatial distribution patterns in the ccRCC microenvironment. Their spatial interplay with immune cells collectively shapes the overall functionality of the TME (30). Finally, the physicochemical properties of the TME, such as the pervasive hypoxic state in the RCC microenvironment, have been shown to directly impair the cytotoxic function of tumor-infiltrating CD8+ T cells; overcoming this hypoxia could potentially enhance their antitumor activity (68, 69). The spatial growth pattern of the tumor itself (for example, superficial versus volumetric growth) also constructs a unique microenvironmental architecture, which in turn influences clonal diversity and intercellular interactions (70).

The intricate interplay and functional reprogramming of immune cell subsets are closely linked. Within the RCC TME, multiple immunosuppressive cell subsets interact with effector T cells to collectively establish an immunosuppressive state. For instance, tumor-associated macrophages (TAMs), MDSCs, and Tregs are key contributors to both primary and acquired resistance to ICIs (64, 71). These cells suppress effector T cell function through diverse mechanisms, including the secretion of inhibitory cytokines (such as IL-10 and TGF-β), expression of immune checkpoint ligands (like PD-L1), and depletion of amino acids essential for T cell activation (such as arginine) (72–74). For example, the enrichment of TAMs in the TME promotes ICI resistance by reducing T cell and NK cell activity, expressing PD-L1, and releasing soluble immunosuppressive factors (75). Further research has uncovered the heterogeneity of TAMs and the impact of their metabolic state on their function; for instance, enhanced glycolysis in TAMs produces lactate, which can drive their polarization toward an M2 (pro-tumoral) phenotype (76, 77). Moreover, in RCC patients undergoing ICI therapy, the frequency of GPNMB-expressing monocytic MDSCs (M-MDSCs) in peripheral blood is significantly higher in patients with progressive disease than in responders, suggesting that specific MDSC subsets may be intimately linked to ICI resistance (59).

The aberrant tumor vasculature and a remodeled extracellular matrix (ECM) also present significant barriers to ICI efficacy. The VEGF pathway not only drives tumor angiogenesis but also contributes to the formation of an immunosuppressive microenvironment. Therefore, combining ICIs with anti-VEGF agents can ‘normalize’ the tumor vasculature, thereby improving perfusion and oxygenation of the TME and facilitating T cell infiltration (78–80). In ccRCC, the spatial interaction between mesenchymal-like tumor cells and a specific CAF subtype, known as myofibroblastic CAFs (myCAFs), is associated with ICI resistance. These CAFs contribute to resistance by creating physical and chemical barriers that restrict immune cell infiltration (81–83).

Metabolic factors must not be overlooked, as the metabolic reprogramming of tumor cells not only supports their growth but also profoundly affects the function of immune cells within the TME. Recent research has highlighted the central role of oxidative phosphorylation (OXPHOS) in ICI resistance in RCC. By analyzing data from multiple clinical trials, including CheckMate, JAVELIN Renal 101, and NCT01358721, as well as clinicopathological data from 25 patients at Tongji Hospital, Tian et al. found that high levels of OXPHOS in tumor tissue, particularly in metastatic lesions, are a significant risk factor for ICI resistance and are associated with poorer PFS and OS in RCC patients (68). Mechanistically, RCC cells with active OXPHOS consume large amounts of oxygen from the TME, exacerbating hypoxia, which in turn suppresses effector T cell function and may induce T cell exhaustion (68). Preclinical studies have confirmed that inhibiting tumor cell OXPHOS can ameliorate the immunosuppressive state of the TME and enhance CD8+ T cell function, thereby increasing the efficacy of anti-PD-L1 therapy (68).

### 2.2 Resistance of ICIs in ccRCC

Although ICIs have markedly improved therapeutic outcomes in ccRCC, acquired or primary resistance remains a key challenge that limits their efficacy (84). A deep understanding of the molecular mechanisms and TME characteristics that drive resistance is crucial for guiding clinical practice and developing novel therapeutic strategies (85). Recent research has built upon this foundation to uncover more profound mechanisms of resistance.

Tumor-cell-intrinsic genetic alterations are a significant contributor to heterogeneous responses and ICI resistance. Alterations in the PBRM1 gene, a common mutation in ccRCC, have been associated with tumor-immune interactions and immunotherapy outcome, although published findings remain conflicting and suggest a context-dependent role (86, 87). Research by Cho et al. further revealed that aberrant alternative splicing of the PBRM1 gene, specifically a reduction in the RBFOX2-mediated exon 26-to-exon 27 splicing variant, is associated with diminished ICI efficacy, offering a new perspective on the role of PBRM1 in this setting (86). In addition, Fan et al. have shown that CDC20-mediated selective autophagic degradation of PBRM1 may influence the effectiveness of immunotherapy in RCC (88). Recent evidence further suggests that the association between PBRM1 mutation and immunotherapy benefit may depend on metabolic subtype, providing a potential explanation for these discordant findings (89). Beyond PBRM1, alterations in other genes are also implicated in the resistance process. Lu et al. found that the expression patterns of endogenous retroviruses (ERVs) correlate with ICI efficacy and could serve as predictive indicators (90). A study by Ma et al. suggested that PTPRZ1 may reduce the efficacy of both TKIs and PD-1 blockade in ccRCC by dephosphorylating and stabilizing RNF26 (12).

The complexity and immunosuppressive state of the TME are critical determinants of ICI efficacy. Disease progression is often accompanied by an evolution of the TME toward an immunosuppressive phenotype. Using single-cell and spatial transcriptomics, Song et al. unveiled the high degree of heterogeneity within the ccRCC TME and identified the SPP1–CD44 signaling pathway as a potential driver of primary resistance to immunotherapy (30). Qiu et al. discovered that the intratumoral infiltration of NKG2A+CD8+ T cells is associated with a poorer prognosis and resistance to immunotherapy in patients with ccRCC; these cells exhibit high expression of checkpoint molecules and impaired effector function (23). Research by Li et al. indicated that TUBA1C might mediate resistance to immune checkpoint blockade by orchestrating an immunosuppressive TME (91). Metabolic dysregulation is another major driver of immunosuppression within the TME. Tian et al. proposed that targeting oxidative phosphorylation (OXPHOS) could enhance the efficacy of combination immunotherapy in renal cancer; for instance, knockdown of Ndufb8 was shown to alleviate the hypoxic exposure of tumor-infiltrating CD8+ T cells and enhance their cytotoxic capacity (68). Zhu et al. explored the potential of using nanoparticle-delivered HIF-2α inhibitors to target hypoxia and inhibit autophagy, thereby augmenting therapeutic effects (69). Systemic factors such as myosteatosis have also been found by Yu et al. to have complex and even contradictory effects on ICI response in patients with metastatic RCC (92). In the circulation, biomarkers such as soluble TIM-3 levels in serum or plasma have been suggested by Pourmir et al. to be predictive of ICI resistance (93). A study by Kato et al. demonstrated that features of the peripheral T-cell receptor repertoire can predict durable responses to anti-PD-1 monotherapy (67). Hwang et al. have also explored the correlation between circulating immune biomarkers and the response to immunotherapy in patients with metastatic RCC (94).

In response to these resistance mechanisms, a variety of strategies are being actively explored. Beyond the widely used combination of ICIs with anti-angiogenic TKIs, novel combination therapy models aim to overcome TME-mediated immunosuppression and adaptive tumor escape (95). These strategies include targeting new immune checkpoints such as TIM-3 and TIGIT, as well as combining ICIs with other immunomodulatory agents, bispecific antibodies, CAR-T cell therapies, therapeutic vaccines, or cytokines (96). For example, Liu et al. investigated the co-delivery of axitinib and PD-L1 siRNA to achieve a synergistic effect of vascular normalization and immune checkpoint inhibition (97). A phase I trial led by Msaouel et al. has been published, evaluating sitravatinib (a multi-kinase TKI targeting TAM receptors) plus nivolumab and ipilimumab in advanced ccRCC; these early-phase data primarily inform feasibility and safety rather than definitive efficacy (98). Research by Wu et al. has shown that the combination of cabozantinib with the mTORC1/2 inhibitor sapanisertib can effectively block multiple resistance mechanisms (99). For patients who experience disease progression after prior ICI therapy, subsequent treatment options include salvage ICI combination therapy (such as nivolumab plus ipilimumab) or switching to targeted agents like belzutifan or everolimus (84, 100).

Furthermore, in the early-stage setting, adjuvant pembrolizumab has established a new standard of care based on the proven efficacy and survival benefits demonstrated in the KEYNOTE-564 trial (101). In stark contrast, other ICI-based strategies have not yielded similar success; specifically, perioperative nivolumab (PROSPER) and adjuvant nivolumab plus ipilimumab (CheckMate 914) have both reported negative results, failing to meet their primary disease-free survival (DFS) endpoints (84, 102).Therefore, the rational design and selection of combination therapies based on a thorough understanding of resistance mechanisms will be a key direction for overcoming ICI resistance in the future.

### 2.3 Clinical research and therapeutic strategy evolution of ICIs for ccRCC

**Figure 2:**
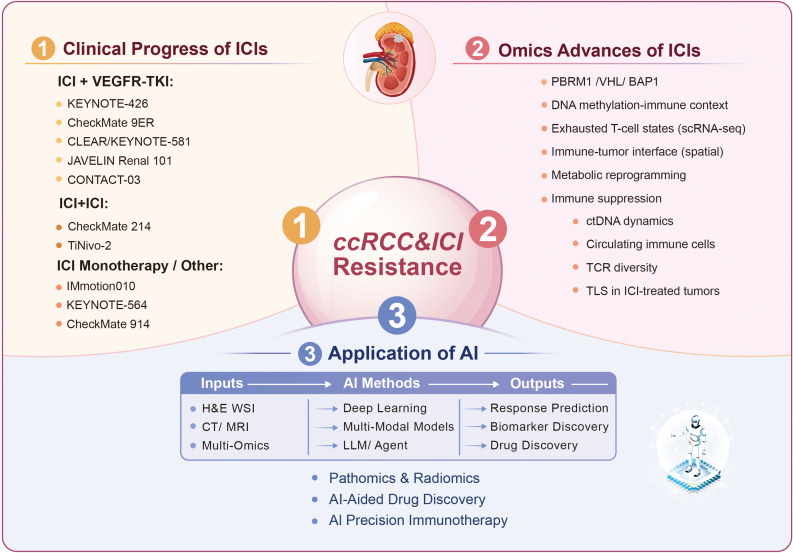
Overview of the major themes covered in this review of ICIs in ccRCC. This schematic summarizes (1) pivotal phase III clinical trials of ICI-based regimens in ccRCC, grouped by combination strategy; (2) key multi-omics advances linked to ICI response and resistance, including genomic/epigenomic features, tumor microenvironment heterogeneity, metabolic and proteomic programs, circulating biomarkers, and immune clonality/tertiary lymphoid structures; and (3) representative AI-enabled workflows integrating pathology images, radiology, and multi-omics data to support response prediction, biomarker discovery, and drug discovery.

ICIs have precipitated a fundamental paradigm shift in the therapeutic landscape of advanced renal cell carcinoma (aRCC), with a particular emphasis on the clear cell histological subtype (ccRCC). Currently, ICI-based synergistic combinations have emerged as the established cornerstone of frontline intervention. Beyond metastatic disease, the strategic integration of ICIs into the perioperative setting has opened novel avenues for optimizing clinical outcomes in localized RCC. This evolution is underpinned by a robust body of evidence from successive clinical investigations, transitioning from monotherapeutic breakthroughs to sophisticated combination strategies (**Figure 2**). A comprehensive synthesis of pivotal clinical trials evaluating these regimens is delineated in **Tables 1**–**4**.

**Table 1:** Summary of key phase III clinical trials of first-line ICI combination therapy for metastatic renal cell carcinoma.

Trial name (registration No.)	Median follow-up (months)	Patient population (key criteria, IMDC risk)	Line of therapy	Sample size (combination/control)	Intervention (drug, dose)	Control (drug, dose)	Primary endpoint(s)	ORR (%) (combo vs control)	CR (%) (combo vs control)	Median PFS (months) (combo vs control) (HR, 95% CI, p-value)	Median OS (months) (combo vs control) (HR, 95% CI, p-value)	Median DOR (months) (combo vs control)	Key ≥grade 3 TRAEs (%) (combo vs control)
ICI + TKI													
KEYNOTE-426 (NCT02853331)	30.6	Advanced ccRCC, any IMDC risk (F: ~31%, I: ~56%, P: ~13%)	First-line	432/429	Pembrolizumab 200mg Q3W + Axitinib 5mg BID	Sunitinib 50mg QD (4/2 wks)	OS, PFS	60 vs 40	9 vs 3	15.4 vs 11.1 (HR 0.71, <0.0001)	47.2 vs 40.8 (HR 0.68, 0.0003)	23.5 vs 15.9	Hypertension (22 vs 20), ALT increase (13 vs 3), Diarrhea (11 vs 5)
CheckMate 9ER (NCT03141177)	18.1 (initial); 67.6 (final)	Advanced ccRCC, any IMDC risk (F: ~23%, I: ~58%, P: ~19%)	First-line	323/328	Nivolumab 240mg Q2W + Cabozantinib 40mg QD	Sunitinib 50mg QD (4/2 wks)	PFS	55.7 vs 27.1 (initial); 55.7 vs 27.4 (final)	8 vs 4.6 (initial); 14 vs NR (final)	16.6 vs 8.3 (HR 0.51, <0.0001) (initial); 16.4 vs 8.3 (HR 0.58) (final)	NR vs NR (HR 0.60, 0.001) (initial); 46.5 vs 35.5 (HR 0.79) (final)	20.3 vs 11.1 (initial); 22.0 vs NR (final)	Hypertension (13 vs 12), Diarrhea (6 vs 5), ALT/AST increase (higher in combo) (initial/final similar)
CLEAR/KEYNOTE-581 (NCT02811861)	26.6 (initial); 49.8 (final OS)	Advanced ccRCC, any IMDC risk (F: ~33%, I: ~57%, P: ~10%)	First-line	355/357	Pembrolizumab 200mg Q3W + Lenvatinib 20mg QD	Sunitinib 50mg QD (4/2 wks)	PFS	71 vs 36 (initial); 71.3 vs 36.7 (at final OS)	16 vs 4 (initial); NR CR (at final OS)	23.9 vs 9.2 (HR 0.39, <0.001) (initial); 23.9 vs 9.2 (HR 0.47) (at final OS)	53.7 vs 54.3 (HR 0.66, 0.005) (initial); 53.7 vs 54.3 (HR 0.79, 0.0424) (final OS)	25.8 vs 14.6 (initial); NR (at final OS)	Hypertension (higher in combo), Diarrhea (higher in combo), Hypothyroidism (higher in combo) (initial/final similar)
JAVELIN Renal 101 (NCT02684006)	11.6 (PD-L1+ initial); 73.7 (Overall pop. final OS)	Advanced ccRCC, any IMDC risk (PD-L1+ ~63%)	First-line	442/444	Avelumab 10mg/kg Q2W + Axitinib 5mg BID	Sunitinib 50mg QD (4/2 wks)	PFS (PD-L1+), OS (PD-L1+)	55.2 vs 25.5 (PD-L1+ initial); 59.7 vs 32.0 (Overall pop. final OS)	NR CR comparison (initial); ≥5-yr DOR rate: 16.4% vs 9.2% (final OS)	13.8 vs 7.2 (HR 0.61, <0.001) (PD-L1+ initial); 5-yr PFS rate: 12.0% vs 4.4% (Overall pop. final OS)	37 vs 44 deaths (PD-L1+ initial); 44.8 vs 38.9 (HR 0.88, 0.067) (Overall pop. final OS)	NR (initial); ≥5-yr DOR rate: 16.4% vs 9.2% (final OS)	Hypertension (higher in combo), Diarrhea (higher in combo), Hypo/hyperthyroidism (higher in combo) (initial/final similar)
ICI + ICI													
CheckMate 214 (NCT02231749) †	25.2 (I/P risk initial); 99.1 (ITT 8-yr)	Advanced ccRCC, IMDC Int/Poor (initial); Any IMDC risk (8-yr ITT)	First-line	550/546 (initial); 550/546 (8-yr ITT)	Nivolumab 3mg/kg + Ipilimumab 1mg/kg Q3W x4, then Nivo Q2W	Sunitinib 50mg QD (4/2 wks)	OS, ORR, PFS (I/P risk initial); OS (ITT 8-yr)	42 vs 26.6 (I/P risk initial); 39.5 vs 33.0 (ITT 8-yr)	9 vs 1 (I/P risk initial); 12.0 vs 3.5 (ITT 8-yr)	11.6 vs 8.4 (HR 0.82, 0.03 (not sig.)) (I/P risk initial); 90-mo PFS rate: 25.4% vs 8.5% (I/P risk 8-yr)	NR vs 26.0 (HR 0.63, <0.001) (I/P risk initial); 52.7 vs 37.8 (HR 0.72) (ITT 8-yr)	NR vs 19.7 (I/P risk initial); 76.2 vs 25.1 (ITT 8-yr)	Immune-related AEs (e.g., rash, colitis, hepatitis, endocrinopathies) more common in combo (initial/8-yr similar)
ICI + ICI + TKI													
COSMIC-313 (NCT03937219) *	45	Previously untreated advanced ccRCC; IMDC intermediate (75%) or poor (25%) risk	First-line	428/427	Cabozantinib 40 mg PO QD + nivolumab 3 mg/kg IV Q3W ×4 + ipilimumab 1 mg/kg IV Q3W ×4, then nivolumab 480 mg IV Q4W (up to 2 years)	Placebo PO QD + nivolumab 3 mg/kg IV Q3W ×4 + ipilimumab 1 mg/kg IV Q3W ×4, then nivolumab 480 mg IV Q4W (up to 2 years)	PFS (BIRC); OS (secondary)	43 vs 36	3 vs 3	16.6 vs 11.2 (HR 0.82, 95% CI 0.69–0.98)	41.9 vs 42.0 (HR 1.02, 95% CI 0.85–1.23; p=0.8366)	NR	81 vs 62 (ALT increase 26–27 vs 6, AST increase 19–20 vs 5, Diarrhea 5 vs 3)

Abbreviations: ALT, alanine aminotransferase; AST, aspartate aminotransferase; BID, twice daily; ccRCC, clear cell renal cell carcinoma; CI, confidence interval; CR, complete response; DOR, duration of response; F, favorable-risk; HR, hazard ratio; I, intermediate-risk; ICI, immune checkpoint inhibitor; IMDC, International Metastatic Renal Cell Carcinoma Database Consortium; ITT, intent-to-treat; I/P, intermediate/poor-risk; TRAEs, treatment-related adverse events; NCT, National Clinical Trial (clinicaltrials.gov registration number); NR, not reached; OS, overall survival; P, poor-risk; PD, progressive disease; PD-L1, programmed death-ligand 1; PFS, progression-free survival; QD, once daily; Q2W, every 2 weeks; Q3W, every 3 weeks; RCC, renal cell carcinoma; SOC, standard of care; TKI, tyrosine kinase inhibitor.

*COSMIC-313 demonstrated a progression-free survival benefit but no overall survival advantage versus nivolumab plus ipilimumab, with a higher rate of grade ≥3 treatment-related adverse events; accordingly, this triplet has not been widely adopted as standard of care.

†Final long-term follow-up further showed that overall survival favored nivolumab plus ipilimumab over sunitinib in the IMDC favorable-risk subgroup, refining earlier analyses in which a clear advantage had not yet been observed.

**Table 2:** Summary of key clinical trials of second-line and subsequent ICI-related therapies for metastatic renal cell carcinoma.

Trial name (registration no.)	Median follow-up (months)	Patient population (key criteria, prior lines & types)	Line of therapy	Sample size (combo/control)	Intervention	Control	Primary endpoint(s)	ORR (%) (combo vs control)	CR (%) (combo vs control)	Median PFS (months) (combo vs control) (HR, 95% CI, p-value)	Median OS (months) (combo vs control) (HR, 95% CI, p-value)	Median DOR (months) (combo vs control)	Key ≥grade 3 TRAEs (%) (combo vs control)
CONTACT-03 (NCT04338269)	15.2	Advanced RCC, ICI-experienced (PD-1/L1 inhibitor as most recent line)	Second/Third-line	263/259	Atezolizumab 1200mg Q3W + Cabozantinib 60mg QD	Cabozantinib 60mg QD	**Negative Study (Did not improve PFS/OS)**	NR ORR comparison	NR CR comparison	10.6 vs 10.8 (HR 1.03, 0.78)	25.7 vs NE (HR 0.94, 0.69)	NR	Serious AEs: 48 vs 33
TiNiVO-2 (NCT04987203)	12.0	Advanced RCC, 1–2 lines ICI-experienced	Second/Third-line	171/172	Tivozanib 0.89mg QD + Nivolumab 480mg Q4W	Tivozanib 1.34mg QD	**Negative Study**	NR ORR comparison	NR CR comparison	5.7 vs 7.4 (HR 1.10, 0.49)	NR OS data	NR	Serious AEs: 32 vs 37
LITESPARK-005 (NCT04195750)	35.8 (Final analysis)	Advanced ccRCC, 1–3 prior lines (incl. PD-(L)1 inhibitor & VEGF-TKI)	Later-line	374/372	Belzutifan 120mg QD	Everolimus 10mg QD	PFS, OS	22.7 vs 3.5	3.5 vs 0	5.6 vs 5.6 (HR 0.75) (24-mo PFS rate: 17.5 vs 4.1)	21.4 vs 18.2 (HR 0.92, 0.18)	19.3 vs 13.7	Anemia, hypoxia (Belzutifan); Stomatitis, rash (Everolimus). Grade ≥3 TRAEs: 39.5 vs 40.0
LITESPARK-003 Cohort 2 (NCT03634540)	24.6 (Early data)	Advanced ccRCC, prior immunotherapy-experienced	Later-line	52 (single arm)	Belzutifan 120mg QD + Cabozantinib 60mg QD	N/A	ORR	31	NR CR	13.8 (Early data)	26.7 (Early data)	NR	Hypertension, diarrhea, fatigue, anemia

Abbreviations same as **Table 1**. AEs, adverse events; N/A, not applicable.

**Table 3:** Summary of key phase III clinical trials of adjuvant/neoadjuvant ICI therapy for renal cell carcinoma.

Trial name (registration no.)	Median follow-up (months)	Patient population (key criteria, risk stratification)	Treatment setting	Sample size (ICI arm/control arm)	Intervention	Control	Primary endpoint	Primary endpoint result (HR, 95% CI, p-value)	Key secondary endpoint result (OS: HR, 95% CI, p-value)	Key ≥grade 3 TRAEs (%) (ICI arm vs control arm)
KEYNOTE-564 (NCT03142334)	24.1 (initial); 57.2 (OS analysis)	ccRCC post-nephrectomy, intermediate-high risk of recurrence (M0 or M1 NED)	Adjuvant	496/498	Pembrolizumab 200mg Q3W (≤17 cycles)	Placebo	DFS	HR 0.68 (0.53-0.87), p=0.002 (initial); HR 0.72 (0.59-0.87) (at OS analysis)	HR 0.62 (0.44-0.87), p=0.005 (at OS analysis)	32.4 vs 17.7 (any cause AEs); 18.9 vs 1.2 (TRAEs) (initial)/32.0 vs 17.7 (any cause AEs) (at OS analysis)
CheckMate 914 Part A (NCT03138512)	37.0	ccRCC post-nephrectomy, high risk of recurrence	Adjuvant	405/411	Nivolumab 240mg Q2W x12 + Ipilimumab 1mg/kg Q6W x4	Placebo	DFS	HR 0.92 (0.71-1.19), p=0.53	OS data immature	38 vs 10 (any cause AEs); Treatment discontinuation rate 32 vs 2
CheckMate 914 Part B (NCT03138512)	27.0	ccRCC post-nephrectomy, high risk of recurrence	Adjuvant	411 (Nivo)/208 (Pbo)/206 (N+I)	Nivolumab 240mg Q2W (≤12 doses)	Placebo	DFS (Nivo vs Pbo)	HR 0.87 (0.62-1.21), p=0.40	OS data immature	Nivo: 17.2; Pbo: 15.0; N+I: 28.9 (any cause AEs)
PROSPER RCC (EA8143) (NCT03055013)	NR	≥T2 or N+ M0 RCC, any histology, planned nephrectomy	Perioperative	~392/~393 (planned 785)	Nivolumab 1 dose pre-op + 9 doses post-op	Surgery + Observation	EFS	Did not improve EFS (trial terminated early)	NR	NR detailed data
IMmotion010 (NCT03024996)	44.7	RCC post-nephrectomy, increased risk of recurrence (≥pT2 or N1, M0)	Adjuvant	390/390	Atezolizumab 1200mg Q3W (16 cycles or 1 year)	Placebo	DFS	HR 0.93 (0.75-1.14), p=0.47	Did not improve OS	20 vs 15 (TRAEs)
RAMPART (NCT03288532)	36 (primary analysis; median follow-up ~3 years)	Resected primary RCC; Leibovich intermediate- or high-risk (score 3–11), including M1 NED; any histology (predominantly clear cell)	Adjuvant	225/340	Durvalumab (13 cycles over 1 year) + Tremelimumab (cycles 1–2 only)	Active monitoring (observation)	DFS	HR 0.65 (0.45–0.93), p=0.0094 (one-sided); 2-year DFS 84% vs 78%	OS data immature/not reported	Grade ≥3 any-cause AEs: 40 vs 8

Abbreviations same as **Table 1** & **2**. EFS, event-free survival; Pbo, placebo; N+I, Nivolumab+Ipilimumab.

**Table 4:** Clinical trial progress of emerging targets and drugs in RCC ICI therapy.

Target name	Drug name/class	Brief mechanism of action	Key RCC-related clinical trials (reg. no., phase)	Combination agent(s) (if any)	Primary RCC study population	Reported key efficacy/safety information	Current development status
LAG-3	Relatlimab (BMS-986016)	Anti-LAG-3 antibody, blocks LAG-3 binding to its ligands, relieves T cell inhibition	FRACTION-RCC (NCT02996110, Phase II)	Nivolumab	IO-naïve advanced RCC	ORR 30%, mDOR 33 wks, Grade 3–4 TRAEs 13%	Results reported
LAG-3	Ieramilimab (LAG525)	Anti-LAG-3 antibody	NCT02460224 (Phase I/II)	Spartalizumab (PD-1 inhibitor)	Advanced solid tumors (incl. RCC)	RCC subgroup data limited	Completed, results pending full publication
TIM-3	Sabatolimab (MBG453)	Anti-TIM-3 antibody	NCT02608268 (Phase I/II)	Spartalizumab (PD-1 inhibitor)	Advanced solid tumors (incl. RCC)	Combination well-tolerated, preliminary anti-tumor activity observed, RCC subgroup data limited	Results reported
TIM-3	INCAGN02390	Anti-TIM-3 antibody	NCT03652077 (Phase I)	Monotherapy or with PD-1 inhibitor	Advanced solid tumors (incl. RCC)	Safety and tolerability assessment	Completed, results pending full publication
TIGIT	Tiragolumab (RG6058)	Anti-TIGIT antibody	NCT03977467 (Phase II); NCT05805501 (DUET-4, Phase II)	Atezolizumab; Tobemstomig + Axitinib	Advanced solid tumors (incl. RCC); First-line ccRCC	Evaluating	Ongoing
TIGIT	Vibostolimab (MK-7684)	Anti-TIGIT antibody	MK-3475-U03 (NCT04626479, Phase Ib/II)	Pembrolizumab	First-line mRCC	Evaluating	Ongoing
ILT4	MK-4830	Anti-ILT4 antibody	NCT04626518 (Phase II)	Pembrolizumab	Advanced solid tumors (incl. RCC)	Combination showed anti-tumor activity, manageable safety	Ongoing
ILT4	CDX-585	Anti-ILT4 bispecific antibody (ILT4 x PD-1)	NCT05788484 (Phase I)	Monotherapy	Advanced solid tumors (incl. RCC)	Evaluating	Ongoing/Recruiting
Tumor Vaccine	Individualized neoantigen vaccine	Induces immune response against tumor-specific neoantigens	NCT02950766 (Phase I); NCT05269381 (Phase I); NCT05641545 (Phase Ib)	Ipilimumab; Pembrolizumab; SOC	ccRCC; Advanced malignancies (incl. RCC); Advanced solid tumors (incl. RCC)	Evaluating safety, immunogenicity, preliminary efficacy	Ongoing/Recruiting
Tumor Vaccine	EVM14	Universal off-the-shelf therapeutic tumor vaccine	Preclinical	ICI	RCC (preclinical)	Preclinical data show enhanced anti-tumor activity with ICI	Preclinical/Planned clinical
CAR-T	CD70-targeted CAR-T	Recognizes and kills CD70-expressing tumor cells	NCT05420519 (Phase I)	Monotherapy	Advanced/metastatic RCC	Evaluating safety and preliminary efficacy	Ongoing/Recruiting
CAR-T	CAIX-targeted CAR-T	Recognizes and kills CAIX-expressing tumor cells	NCT04969354 (Phase I)	Monotherapy	Advanced/metastatic RCC	Evaluating safety and preliminary efficacy	Ongoing/Recruiting
CAR-NK	CD70-targeted CAR-NK	Recognizes and kills CD70-expressing tumor cells	NCT05703854 (Phase I/II)	Fludarabine + Cyclophosphamide (lymphodepletion)	Advanced solid tumors (incl. RCC)	Evaluating safety and preliminary efficacy	Ongoing/Recruiting

Abbreviations as in previous tables. IO, immuno-oncology; SOC, standard of care.

The landmark CheckMate 025 trial, which established the efficacy of nivolumab monotherapy in the second-line setting, catalyzed the exploration of immune checkpoint inhibitors (ICIs) in treatment-naïve populations (18). Consequently, the therapeutic paradigm for advanced/metastatic ccRCC (a/mccRCC) has undergone a definitive transition from TKI monotherapy to ICI-based combination regimens. Current international guidelines now endorse ICI-ICI or ICI-tyrosine kinase inhibitor (TKI) combinations as the preferred standard of care, predicated on their demonstrated superiority in prolonging progression-free survival (PFS) and overall survival (OS), alongside robust objective response rates (ORR), relative to the former benchmark, sunitinib (103). This paradigm shift not only reflects the clinical maturity of immunotherapeutic strategies but also redefines the long-term survival expectations for patients with advanced renal malignancy.

#### 2.3.1 ICI–ICI combination therapy

The CheckMate 214 study established the role of nivolumab plus ipilimumab as a first-line treatment for patients with intermediate- and poor-risk a/mccRCC, as defined by the International Metastatic RCC Database Consortium (IMDC) criteria (104). Long-term follow-up data reported by Motzer R.J. et al. show a durable OS benefit for this combination in the intermediate/poor-risk population, with a median OS of 47.0 months versus 26.6 months for the sunitinib arm (HR 0.69) and a complete response (CR) rate of approximately 11% (17, 104, 105). Notably, nearly 8-year follow-up data reported by Tannir N.M. et al. revealed that even among patients with IMDC favorable-risk disease, overall survival favored nivolumab plus ipilimumab over sunitinib, whereas earlier analyses had not shown a clear advantage, and the PFS data favored sunitinib (104, 106).

#### 2.3.2 ICI–TKI combination therapy

The combination of ICIs with VEGFR-TKIs represents another successful first-line treatment strategy, with several ICI-TKI regimens having now received approval.

Pembrolizumab plus Axitinib (KEYNOTE-426 study): The KEYNOTE-426 study, reported by Rini B.I. et al., confirmed the significant survival benefit of pembrolizumab plus axitinib over sunitinib in treatment-naive patients with a/mccRCC (107, 108). According to nearly 5-year follow-up data published by Rini B.I. et al., the median OS was 47.2 months in the combination arm versus 40.8 months in the sunitinib arm (HR 0.79); the 5-year OS rates were 41.7% and 37.3%, respectively; median PFS was 15.7 months versus 11.1 months (HR 0.69) (66, 104, 109–112). A subgroup analysis of the East Asian population (median follow-up of 42.8 months) also showed a consistent efficacy trend, although the confidence interval for the OS HR was wide (113).

Nivolumab plus Cabozantinib (CheckMate 9ER study): The results of the CheckMate 9ER study confirmed the superiority of nivolumab plus cabozantinib over sunitinib for the first-line treatment of aRCC (114–116). Its 3-year follow-up data showed a median OS of 49.5 months in the combination arm versus 35.5 months in the sunitinib arm (HR 0.70); median PFS was 16.6 months versus 8.4 months (HR 0.58), with an ORR of 55.7% and a CR rate of 12.4% (114–116).

Pembrolizumab plus Lenvatinib (CLEAR/KEYNOTE-581 study): The CLEAR study, reported by Motzer R.J. et al., demonstrated that first-line treatment with pembrolizumab plus lenvatinib led to significant improvements in PFS, OS, and ORR compared to sunitinib in patients with aRCC (117, 118). This combination achieved a median PFS of 23.9 months, an ORR of 71.3%, and a CR rate of 18.3%, making it one of the ICI-TKI regimens with the most impressive response depth and PFS reported to date (117–119). Although the final analysis reported numerically similar median OS for the combination and sunitinib arms (53.7 vs. 54.3 months), an HR of 0.86 confirmed a sustained survival benefit for the combination regimen, capturing the overall reduction in mortality risk beyond a single-point median estimate. In the final prespecified OS analysis of the study (median follow-up of 50.1 months), the median OS was 53.7 months for the pembrolizumab plus lenvatinib arm versus 54.3 months for the sunitinib arm (HR 0.86) (117–119).

Limitations of cross-trial comparisons. Apparent differences in ORR, PFS, and OS across first-line ccRCC trials should not be interpreted as evidence of comparative superiority. Cross-trial comparisons are confounded by heterogeneity in eligibility criteria, IMDC risk distribution, baseline disease burden (including sarcomatoid features), PD-L1 assays and cutoffs, endpoint definitions and assessment schedules, follow-up duration, and access to subsequent therapies. Therefore, regimen ranking requires head-to-head studies or appropriately adjusted comparative analyses rather than unanchored numerical comparisons.

#### 2.3.3 IMDC risk stratification

The IMDC risk model is a crucial tool for guiding first-line treatment decisions in advanced RCC (104, 120). For patients with favorable-risk disease, ICI-TKI combinations are the standard of care recommended by current guidelines (104, 120). Although long-term follow-up from CheckMate 214 showed that OS favored nivolumab plus ipilimumab in the favorable-risk group, ICI-TKI combinations remain more commonly selected in this setting because of their disease-control advantages, including higher response rates and more favorable PFS outcomes in this subgroup (17, 104, 105). For patients with intermediate- or poor-risk disease, both nivolumab plus ipilimumab and various ICI-TKI combinations are recommended as first-line options by authoritative guidelines such as the NCCN and ESMO (121, 122). At present, ICI-TKI regimens have become a more broadly applicable first-line choice in most scenarios owing to their generally higher ORRs and longer median PFS (117–119). Nevertheless, nivolumab plus ipilimumab retains an important role, delivering deep and durable responses in a subset of patients, with long-term follow-up also showing an OS benefit in selected favorable-risk patients (104, 121, 122).

#### 2.3.4 Triplet therapy

To further enhance efficacy, investigators have explored triplet therapies. The COSMIC-313 study, reported by Choueiri T.K. et al., evaluated the addition of cabozantinib to the nivolumab plus ipilimumab backbone (cabo+nivo+ipi) as a first-line treatment for patients with IMDC intermediate- or poor-risk aRCC (123). The study data showed that the triplet therapy significantly improved PFS compared to placebo plus nivo+ipi (median not reached vs. 11.3 months; HR 0.73). However, according to published data with longer follow-up, there was no significant difference in OS, and the rate of grade 3/4 treatment-related adverse events (TRAEs) was higher in the triplet arm (81% vs. 62%), which challenges its future clinical application (123).

#### 2.3.5 Perioperative ICI therapy in RCC

Adjuvant therapy aims to reduce the risk of postoperative recurrence in patients with high-risk localized RCC. The breakthrough results from the KEYNOTE-564 study represent a milestone in this field. Findings from Choueiri T.K. et al. and Powles T. et al. showed that at a median follow-up of 57.2 months, adjuvant pembrolizumab significantly improved both DFS (HR 0.72) and OS (HR 0.62) compared to placebo, establishing it as the standard of care in this population (101, 124, 125). In contrast, other adjuvant therapy trials, such as IMmotion010 (atezolizumab), CheckMate 914 (nivolumab +/- ipilimumab), and PROSPER RCC (perioperative nivolumab), failed to meet their primary endpoints (126–129).

The theoretical advantage of neoadjuvant ICI therapy lies in leveraging the *in situ* tumor to elicit a more robust immune response (84, 130). Published data thus far are mainly from small phase I/II clinical trials, which have shown that neoadjuvant ICI monotherapy typically yields a low ORR and rare pathological complete responses (pCRs) (84, 130). Beyond ICI-based approaches, the VEGF-TKI cabozantinib has also demonstrated clinical activity and safety as a targeted neoadjuvant option for locally advanced nonmetastatic ccRCC (131).

However, optimizing perioperative treatment strategies is a complex issue, and neoadjuvant therapy faces challenges such as potential surgical delays and determining how to best assess pathological response (132).

### 2.4 Revealing new dimensions in renal cell carcinoma immunotherapy mechanisms with multi-omics technologies

The application of multi-omics technologies has provided crucial data and novel perspectives for systematically understanding the heterogeneity of treatment response and the mechanisms of resistance to ICIs in ccRCC, driving the field from phenomenological observation toward mechanism-driven, personalized therapeutic exploration (30) (**Figure 2**).

Genomic and epigenomic alterations form the foundation influencing the response of RCC to ICI therapy. Sequencing analysis of the tumor genome has revealed associations between specific mutations and ICI efficacy. For instance, research by Deutsch et al. suggests that integrating tumor histological features with the mutational status of genes such as PBRM1 could lead to the construction of more effective models for predicting ICI efficacy (133). Furthermore, a study by Hagiwara et al. indicates that in PBRM1-mutant ccRCC, low expression of DNA damage repair (DDR) pathway genes, such as PARP1, may predict better overall survival with PD-L1 inhibitors (134). To provide a balanced molecular overview, BAP1 mutations define a distinct, aggressive proliferative subtype in ccRCC and have been reported to be largely mutually exclusive with PBRM1, supporting divergent molecular trajectories (135). Consistently, integrated multi-omics analyses from IMmotion151 suggest that proliferative transcriptional programs may associate with differential outcomes across VEGF-targeted therapy versus ICI-based combinations, underscoring the need to interpret single-gene markers within broader molecular context (66).At the epigenomic level, chromatin accessibility maps generated through techniques like ATAC-seq, as shown in studies by Yu et al. and Lu et al., help identify non-coding RNAs (lncRNAs) and cis-regulatory elements that control key biological behaviors in ccRCC, such as invasion and migration, providing a basis for understanding the epigenetic mechanisms of gene expression regulation (136, 137).

Transcriptomic analyses, particularly the integrated application of single-cell RNA sequencing (scRNA-seq), spatial transcriptomics, and immune repertoire sequencing, have profoundly deepened our understanding of cellular heterogeneity, immune dynamics, and spatial organization within the RCC TME. This has provided core insights for elucidating ICI mechanisms of action and discovering new biomarkers. The decisive role of TME cellular composition, functional states, and their spatial distribution in ICI efficacy is a current research hotspot. scRNA-seq technology has successfully identified several new or critical cell subpopulations within the RCC TME. For example, a study by Ge et al. found a significant enrichment of a specific APOE-positive macrophage population with an M2-like immunosuppressive phenotype in ICB-resistant ccRCC; the proportion of these cells was closely associated with poor prognosis and inferior ICB efficacy. Tumor cell-secreted SPP1 was found to recruit and polarize these macrophages, which in turn suppressed T cell function by releasing TGF-β. Concurrently, high expression of CEBPD in these macrophages was also linked to immunosuppression (138). Research by Krishna et al., using multi-regional scRNA-seq analysis, revealed a strong association between the gene expression signatures of tissue-resident memory T cells (Trm) and specific TAM subpopulations with tumor topology and ICI reactivity (139). Meanwhile, a study by Davidson et al. showed that mesenchymal-like tumor cells and myofibroblastic cancer-associated fibroblasts (myoCAFs) in ccRCC, by virtue of their unique gene expression profiles and interactive signals, are closely linked to disease progression and immunotherapy response, collectively highlighting the critical impact of tumor and stromal cell heterogeneity on ICI efficacy (81). The foundational work by Bi et al. systematically mapped the dynamic reprogramming of tumor cells and various immune cells within the TME of advanced RCC patients during immunotherapy, providing an early single-cell perspective for understanding ICI mechanisms of action and resistance (140). Together, these studies underscore the pivotal role of scRNA-seq in identifying key cellular players and their interaction networks within the TME, especially in revealing the core contribution of macrophage heterogeneity and tumor-stromal interactions to ICI resistance mechanisms.

Spatial transcriptomics further enables the *in-situ* study of these cellular states. Raghubar et al. discovered that the TME of high-grade ccRCC is enriched with immune cells exhibiting exhausted or pro-tumoral phenotypes, yet these cells do not consistently overexpress classic immune checkpoint genes. Moreover, specific types of monocytes were found to spatially envelop exhausted CD8+ T cells and highly express TIM-3 and LAG-3 (141). Kinget et al., by integrating spatial multi-omics data, identified specific spatial interaction patterns between pro-inflammatory TAMs and exhausted CD8+ T cells in ICB-responsive advanced RCC patients. This was associated with the expression of favorable HLA alleles, and an HLA-related gene expression signature constructed on this basis could effectively predict ICB efficacy (142). These findings highlight the critical influence of the spatial arrangement of TME cells on the effects of ICIs.

The role of clonal evolution of the adaptive immune response and organized structures, such as tertiary lymphoid structures (TLS), in ICI efficacy is gaining increasing attention. Immune repertoire sequencing revealed in the ADAPTeR study by Au et al. that the response of mRCC patients to nivolumab was significantly correlated with the number of pre-expanded TCR clones before treatment and the sustained maintenance of these clonal clusters after treatment, emphasizing the importance of “pre-existing immunity” (39). A study by Meylan et al., through spatial analysis, found that TLS within RCC tumors are key sites for *in-situ* B cell maturation and antibody production, and that IgG-coated tumor cells are associated with better responses to ICI therapy and longer PFS (83). Subsequent studies have further confirmed that high-density, mature TLS are associated with favorable prognosis and treatment reactivity in ccRCC patients, and that the spatial location and maturation state of TLS within the tumor are crucial for their function (143, 144). These findings suggest that B cell-mediated humoral immunity and TLS may play a previously underestimated key role in antitumor immunity. Bulk RNA sequencing, combined with advanced algorithms, continues to provide important information for discovering molecular mechanisms and gene expression profiles associated with ICI treatment response. By analyzing bulk RNA sequencing data from multiple ICI clinical trials, Tian et al. found that high levels of an oxidative phosphorylation (OXPHOS) signature in tumor cells were a risk factor for poor ICI treatment outcomes in RCC patients, a mechanism likely mediated by its effects on TME hypoxia and T cell function (68).

Proteomics and metabolomics offer direct evidence of the functional output of RCC’s response to ICI therapy, and existing studies have identified several circulating metabolites or pathway alterations associated with ICI efficacy. For example, research by Li et al. showed that an elevated kynurenine/tryptophan (Kyn/Trp) ratio in the blood during ICI therapy was associated with poorer survival prognosis for patients (145). Combined with the aforementioned findings by Tian et al. from the transcriptomic level on the role of the OXPHOS pathway in ICI resistance (68), this points toward “metabolic checkpoints” as a key factor mediating ICI resistance. These findings suggest that circulating metabolites have potential as dynamic, non-invasive biomarkers. Similarly, in the field of proteomics, the analysis of proteins in circulating plasma is becoming an important direction for exploring biomarkers of ICI treatment response. For instance, a study by Montemagno C. et al. found that the levels of soluble PD-1 (sPD-1) and PD-L1 (sPD-L1) in the plasma of mRCC patients might be related to the efficacy of different treatment regimens (146). Maritaz et al. observed that higher concentrations of IL-8 and LDH were associated with overall survival (19); Schoenfeld et al. proposed that circulating IL-18BP levels and their changes could serve as potential predictive biomarkers for ICI efficacy (10); and Pourmir et al. found that soluble TIM-3 was predictive of resistance (93). Furthermore, liquid biopsy analysis of protein components in extracellular vesicles such as exosomes (e.g., proteins like CA9, CP, MMP9, DKK4, PODXL in urine exosomes) is also being explored for its potential application as a non-invasive biomarker for diagnosing and predicting ICI response (147).

These in-depth studies based on multiple omics levels have provided key insights into the mechanisms of response and resistance to ICI therapy in RCC, driving the development of precision immunotherapy strategies for this disease.

### 2.5 AI-driven immunotherapy with ICIs for renal cell carcinoma

Artificial intelligence (AI), particularly machine learning (ML) and deep learning (DL) algorithms, is emerging as a key technology to propel the treatment of RCC with ICIs into an era of personalized precision medicine, owing to its exceptional capabilities in processing high-dimensional complex data, identifying subtle patterns, and constructing predictive models (148, 149). Traditional biomarkers such as PD-L1 expression and tumor mutation burden (TMB) have shown limited utility in predicting ICI efficacy in RCC, failing to meet the urgent clinical need for accurate predictive tools (150, 151). By integrating multi-dimensional information, including radiomics, pathomics, genomics, and clinical data, AI holds the promise of achieving breakthroughs in several areas, including the discovery of novel biomarkers and the prediction and management of treatment efficacy and prognosis (**Figure 2**).

Kidney injury molecule-1 (KIM-1) has emerged as a particularly promising circulating biomarker in ccRCC, although its current clinical use has largely been explored through relatively simple stratification approaches. A biomarker analysis from the phase III IMmotion010 adjuvant trial showed that higher post-nephrectomy plasma KIM-1 was associated with increased recurrence risk, and that longitudinal KIM-1 kinetics tracked disease relapse; notably, treatment effect estimates for adjuvant atezolizumab versus placebo varied across KIM-1–defined subgroups, supporting KIM-1 as a candidate marker of minimal residual disease and potential treatment selection (152). Integrating serial KIM-1 measurements with radiomics, pathomics, multi-omics, and clinical variables may provide a more appropriate setting for AI-enabled risk stratification, particularly when nonlinear interactions, longitudinal changes, and multivariable patterns need to be modeled beyond simple dichotomized cutoffs.

Digital pathology, especially when combined with AI algorithms for whole-slide imaging (WSI), has given rise to the emerging field of pathomics, which aims to high-throughput extract and analyze a vast amount of quantifiable morphological and spatial features from WSIs (153–155). Studies have demonstrated that AI performs exceptionally well in the diagnosis, histological subtyping, and grading of RCC. For instance, ML models based on handcrafted features can distinguish between different grades of clear-cell RCC (ccRCC) (156, 157). Furthermore, models based on deep neural networks can accurately differentiate various RCC subtypes and perform ISUP nuclear grading for ccRCC (156–158). Beyond basic diagnosis and grading, AI models have been employed to directly predict the prognosis and survival of patients with ccRCC from hematoxylin and eosin (H&E) stained pathology images (156, 157). Wessels et al. showed that a DL model could directly predict overall survival (OS) from H&E images, demonstrating significant predictive value in multivariate analysis (159). Importantly, pathomics has also shown promise in predicting response to ICI therapy. AI-driven spatial analysis tools can segment and quantify tumor-infiltrating lymphocytes (TILs) and classify the TME into different immune phenotypes (such as inflamed, immune-excluded, and immune-desert) based on the spatial distribution of TILs; these phenotypes are significantly associated with objective response rate (ORR) and progression-free survival (PFS) following ICI treatment (32). Although these studies have primarily focused on non-small cell lung cancer (NSCLC), similar AI-driven spatial analysis methods are equally applicable to RCC for assessing the immune cell composition and spatial architecture of the TME, which is crucial for predicting ICI response (160). Moreover, the Lunit SCOPE IO deep learning model, developed by Park et al., can automatically analyze H&E-stained WSIs to classify tumors by immune phenotype (for example, inflamed immunophenotype, IIP) (32). Another significant advance in pathomics is the use of AI to predict the molecular features of tumors from histological images, such as gene mutation status. In other cancer types, AI has successfully predicted various gene mutations (BRAF, KRAS, NRAS, and PIK3CA in colorectal cancer, and ALK, ROS1, and TP53 in lung cancer) (161, 162). Developing similar AI models to predict specific ICI-driving gene mutations (such as PBRM1, BAP1, and SETD2) from H&E images of ccRCC is technically feasible and biologically plausible. The development of such predictive models would facilitate the acquisition of information on tumor molecular heterogeneity at the histological level, further refining the selection of patients for ICI therapy.

Radiomics involves the high-throughput extraction of a large number of quantitative features from standard medical images (such as CT, MRI, and PET) using advanced algorithms. These features are then used to build mathematical models to non-invasively reveal tumor biology, predict treatment response, and determine patient prognosis (163–165). In the context of ICI therapy for RCC, the potential of radiomics as a non-invasive tool for predicting efficacy and characterizing tumors is increasingly apparent. In recent years, several studies have explored the use of radiomic features from pre-treatment CT or ultrasound images to predict the response of RCC patients to ICI therapy. For example, Khene et al. investigated the potential of radiomics in predicting the response of patients with metastatic RCC (mRCC) to nivolumab treatment (166). Furthermore, Rossi E et al. analyzed baseline CT images of patients with advanced RCC undergoing ICI therapy and found that several first-order statistical radiomic features were significantly associated with disease progression, suggesting that quantitative features from baseline CT images could be used to identify patients with a poor response to ICIs at an early stage (167). Duwe G et al. utilized a fully automated AI segmentation technique to assess changes in spleen volume, finding that a smaller spleen volume early after treatment initiation was associated with improved OS in patients with advanced RCC, thus providing a new perspective for monitoring ICI efficacy (168). These studies indicate that radiomics can capture information on tumor heterogeneity that is imperceptible to the human eye, which may indirectly reflect the internal biological properties of the tumor that are related to ICI efficacy. However, the clinical translation of radiomics research still faces challenges, including the standardization of image acquisition, the robustness of feature extraction, model reproducibility, and multi-center external validation. Future research will need to more strictly adhere to standardized protocols and validate the clinical utility of these models in large-scale prospective cohorts (169, 170).

Multi-faceted approaches that integrate computational and experimental strategies are accelerating the discovery of novel immunotherapy drugs. The combination of AI and high-throughput screening enables the analysis of vast chemical and biological datasets, thereby efficiently identifying compounds with potential immunomodulatory properties (171, 172). Advanced screening platforms, such as those that mimic the cancer immune microenvironment or utilize co-culture systems, can simultaneously test numerous compounds to discover small-molecule immunomodulators that enhance antitumor immune responses (173–176). For instance, the High-Throughput Immunomodulator Phenotypic Screening Platform (HTiP) has successfully identified inhibitors of apoptosis proteins (IAPs) as potential anticancer immune enhancers (173, 174). Complementing these methods, “drug repurposing” strategies, which screen libraries of approved drugs for previously unrecognized immunomodulatory effects, offer a time- and cost-effective approach; the discovery of the inhibitory effect of Adefovir Dipivoxil on T-cell proliferation is one such example (177). In addition, machine learning algorithms can leverage public bioactivity data to predict drug-target interactions and generate hypotheses, thereby optimizing the prioritization of candidate drugs for further investigation (178, 179).

Beyond drug discovery, AI plays a pivotal role in personalizing immunotherapy based on individual patient characteristics to maximize efficacy. AI algorithms can analyze complex genomic and transcriptomic datasets to guide precision therapy, for instance, by predicting the most effective ICI therapy for a patient (180–182) A core focus of this immunogenomic approach is the use of AI-driven techniques to identify tumor-specific neoantigens, which is crucial for developing personalized vaccines and targeted therapies that can leverage a patient’s unique immune response (183–187). To more comprehensively characterize tumors and more accurately predict ICI efficacy, the integrated analysis of multi-omics data—including genomics, transcriptomics, and proteomics—is emerging as a powerful strategy. AI, particularly deep learning algorithms, possesses a natural advantage in processing and integrating such high-dimensional, heterogeneous data. For example, the COMPASS model utilizes a Transformer and a “concept bottleneck” design to predict ICI treatment response by mapping the tumor transcriptome to a layer of biologically interpretable immune concepts (188). Similarly, a multi-omic integrative analysis of patients with ccRCC identified three molecular subtypes with distinct molecular features, clinical prognoses, and varying sensitivities to different therapies, including PD-1 inhibitors (189). Other advanced models, such as MutFormer, a Transformer-based tool, can be used to predict the pathogenicity of missense mutations, thereby deepening our understanding of the tumor genetic landscape for therapeutic targeting (190). Although multi-omics integration and large models still face challenges related to data acquisition, heterogeneity processing, and model interpretability, they are undoubtedly paving the way for the realization of individualized precision ICI therapy (191–193).

## 3 Conclusions and perspectives

The treatment landscape of advanced ccRCC has been reshaped by ICI-based combination therapies, which are now the standard of care. However, the efficacy of these transformative treatments is profoundly limited by primary and acquired resistance, stemming from the complex interplay between tumor-intrinsic factors and an immunosuppressive tumor microenvironment.

As detailed in this review, the convergence of multi-omics technologies and artificial intelligence (AI) is providing unprecedented insights into this heterogeneity. AI-powered analysis of pathology (pathomics) and radiology (radiomics) is yielding promising non-invasive biomarkers, while deep learning is beginning to integrate multi-modal data to predict therapeutic outcomes. Looking forward, the next frontier will involve the prospective validation of these AI-driven models in large clinical cohorts to ensure their reproducibility and utility. Unraveling resistance mechanisms will continue to fuel the rational design of novel combination therapies, while harnessing liquid biopsies for dynamic monitoring may guide therapy in real time. Ultimately, integrating these data streams holds the key to moving beyond empirical treatment and realizing the full potential of personalized immuno-oncology for every patient with ccRCC.
